# Exercise During Pregnancy: Obstetricians’ Beliefs and Recommendations Compared to American Congress of Obstetricians and Gynecologists’ 2015 Guidelines

**DOI:** 10.7759/cureus.3204

**Published:** 2018-08-25

**Authors:** Lucas D McGee, Carly A Cignetti, Amelia Sutton, Lorie Harper, Candice Dubose, Sara Gould

**Affiliations:** 1 School of Medicine, University of Alabama at Birmingham, Birmingham, USA; 2 Obstetrics and Gynecology, Maternal Fetal Medicine, Novant Health, Birmingham, USA; 3 Obstetrics and Gynecology, University of Alabama at Birmingham, Birmingham, USA; 4 Orthopedic Surgery, University of Alabama at Birmingham, Birmingham, USA; 5 Orthopedic Surgery, University of Alabama, Birmingham, USA

**Keywords:** exercise, pregnancy, guidelines, recommendations, aerobic, strength training, survey

## Abstract

Purpose

Obesity and excessive weight gain during pregnancy is a growing problem, conferring severe health risks for both mother and fetus. Exercise can help combat this epidemic. However, many pregnant women are not meeting the American Congress of Obstetricians and Gynecologists’ (ACOG’s) 2015 guidelines for exercise during pregnancy. The objective of this study was to evaluate obstetricians’ beliefs and recommendations regarding exercise during pregnancy compared to ACOG’s 2015 recommendations.

Method

Obstetricians were recruited via three different forums to complete a twenty-question survey: at a regional conference for Alabama and Mississippi ACOG members, at the University of Alabama at Birmingham’s Obstetrics and Gynecology Department’s Grand Rounds, and via telephone. Univariate statistical analysis was conducted with RedCap.

Results

Seventy-one surveys were completed: 33 from the ACOG conference, 27 from Grand Rounds, and 11 from those recruited by telephone. Eighty-eight percent (n=60) of respondents correctly identified ACOG’s recommendation of unrestricted exercise for women with uncomplicated pregnancies. One-fourth (24%; n=16) regularly discuss exercise with most (76%-100%) pregnant patients. Most (57%; n=59) do not consistently (“never,” “rarely,” or “sometimes”) recommend sedentary patients begin exercising during pregnancy. Nearly all (97%; n=66) advise first-trimester patients to perform aerobic exercise two to five days per week, but the recommended duration varies. One-fourth (24%; n=16) do not recommend strength-training exercise during the first trimester. Twenty-five percent (n=17) and 32% (n=22) recommend decreased aerobic or strength-training exercise, respectively, in the third trimester. More than half (54%; n=37) recommend pregnant patients limit exercise by heart rate, most commonly 121-140 bpm (25%; n=17) or 141-160 bpm (24%; n=16). Sixty-eight percent (n=46) feel “comfortable” or “very comfortable” providing advice on exercise during pregnancy.

Conclusion

Despite believing exercise benefits pregnant women, knowing ACOG’s 2015 guidelines endorse unrestricted exercise for women with uncomplicated pregnancies, and feeling comfortable discussing this topic with patients, obstetricians are not consistently counseling their pregnant patients on exercise. Notably, physicians are not instructing their sedentary pregnant patients to exercise. While most physicians provide appropriate advice on aerobic exercise, their advice on resistance training, maximum heart rate during exercise and third-trimester exercise are often discordant with ACOG’s guidelines.

## Introduction

The American Congress of Obstetricians and Gynecologists (ACOG) Committee on Obstetric Practice revised its January 2002 recommendations regarding exercise during pregnancy and the postpartum period and published its most recent opinion in December 2015. Whereas the 2002 ACOG Committee Opinion advises >30 minutes of aerobic exercise on most days of the week, the new guidelines recommend women with no medical contraindications perform at least 20 to 30 minutes per day of aerobic and strength-conditioning exercise before, during, and after pregnancy. Moreover, the updated guidelines state obese pregnant women and those who were sedentary before pregnancy should be encouraged to gradually adopt an exercise program, whereas ACOG did not previously specify recommendations for these patients. Last, the 2015 guidelines expand upon the 2002 statement that exercise may help prevent gestational diabetes mellitus (GDM), stating exercise may help prevent pre-eclampsia and cesarean and operative vaginal delivery, and decrease postpartum recovery time [1].

In 2006, Entin and Munhall surveyed obstetricians about their beliefs and recommendations regarding exercise during pregnancy and compared their responses to the 2002 ACOG Guidelines [2-3]. Discordant with ACOG’s recommendations, they found a significant percentage of obstetricians do not routinely advise healthy, pregnant patients to exercise, and they seldom instruct sedentary pregnant patients to begin an exercise program. Subsequent studies provide similar findings of physicians not regularly or accurately counseling their pregnant patients about exercise [4-5], particularly those who were sedentary before pregnancy [6]. This is an issue because many pregnant women are not meeting ACOG’s exercise recommendations, or not exercising at all; [7-8] but women may be more likely to exercise during pregnancy if their obstetricians encourage them [9]. Considering the 2015 ACOG guidelines on exercise during pregnancy, and the pertinence of this topic given the current obesity epidemic, we aim to provide an updated survey of obstetricians’ beliefs and recommendations regarding exercise during pregnancy.

## Materials and methods

The research study was approved by the University of Alabama at Birmingham’s Institutional Review Board (IRB). Participants were eligible if they were a licensed physician or nurse practitioner who provides care for women during pregnancy. Study participants were recruited from the 2016 Joint Annual Spring Meeting for Alabama and Mississippi ACOG members in Sandestin, Florida in May 2016. All conference attendees were given the option to complete the survey without incentive or reward. All participants provided informed consent, and no identifying information was collected. Questionnaires were collected in a drop box in the meeting lobby. To increase the sample size, additional participants were recruited from the University of Alabama at Birmingham’s Obstetrics and Gynecology Department’s Grand Rounds in Birmingham, Alabama in July 2016. Additionally, obstetricians practicing in Birmingham and Fort Payne, AL were recruited via telephone, and their completed surveys were collected in person by a study investigator through November 2016.

The survey consisted of twenty multiple choice questions and is available in the Appendix. Thirteen questions are based upon an eighteen-item survey published by Entin and Munhall in 2006 [4], and seven questions are original. Of these, four questions are self-descriptive (e.g., role caring for pregnant patients, clinical title, years of experience, gender); one question measures the participants’ knowledge of ACOG’s most recent guidelines regarding exercise during pregnancy; eleven questions ask about specific recommendations given to pregnant women regarding exercise during pregnancy; three questions ask the provider’s belief regarding the health implications of exercise during pregnancy and need for future research on this topic; and one question inquires about the provider’s comfort level providing advice on exercise during pregnancy. The survey was reviewed and validated by two academic obstetricians. Survey responses were recorded in research electronic data capture software (REDCap) which was used to conduct univariate statistical analysis.

## Results

Provider characteristics

One hundred and twenty-one providers were contacted to participate in the study: 70 at the ACOG conference, 40 at the UAB Obstetrics and Gynecology Department's grand rounds, and 11 by telephone. Of these, 71 participants completed the survey: 33 from the conference, 27 from grand rounds, and 11 contacted by telephone. Three participants were excluded for indicating they no longer provide obstetric care. Sixty-eight participants indicated they were active providers of pregnant women, giving a n=68 sample size for all questions and a 57% overall response rate for the study. All respondents indicated they were physicians, most (53%; n=36) with less than five years of experience and most (62%; n=42) female. Provider characteristics are displayed in Table 1.

**Table 1:** Provider characteristics (n=68)

Provider title	%	n
Nurse Practicioner	0	0
Physician	100	68
Years practicing obstectrics		
<5	52.9	36
6-10	13.2	9
11-15	5.9	4
16-20	8.8	6
>20	19.1	13
Gender		
male	38.2	26
female	61.8	42

Initiation of discussion

Physicians were surveyed about the percent of their pregnant patients with whom exercise is discussed, and whether they, the patient or another staff member, typically initiates this discussion (Table 2). The percent of pregnant patients with whom exercise is discussed is distributed near evenly across four quartiles; only one-fourth (24%; n=16) of respondents discuss exercise with the majority (76%-100%) of patients. Just over half (56%; n=38) initiate this discussion themselves, and 41% (n=28) state the patient initiates this conversation. The majority (57%; n=39) either “never,” “rarely,” or only “sometimes” recommend their sedentary pregnant patients begin exercising (Table 3).

**Table 2:** Percent of patients with whom exercise during pregnancy was discussed and initiator of discussion

Percent of patients counseled	%	n
0-25%	23.5	16
26-50%	27.9	19
51-75%	25.0	17
75-100%	23.5	16
Initiator of discussion		
no one	1.5	1
physician	55.9	38
patient	41.2	28
staff	1.5	1

**Table 3:** Frequency with which providers recommend sedentary patients initiate exercise during pregnancy

Frequency	%	n
never	8.8	6
rarely	20.6	14
sometimes	27.9	19
usually	30.9	21
always	11.8	8

Advice regarding aerobic and strength-conditioning exercise

Physicians were surveyed about the frequency (days per week) and/or duration (minutes per session) for which they advise pregnant women to perform aerobic (e.g., stationary biking, jogging, swimming, etc.) and strength-conditioning exercise when discussing exercise during the first trimester (Table 4). Nearly all physicians (97%; n=66) report they advise their first-trimester patients to perform aerobic exercise two to three or four to five days per week, but the recommended duration varies, with physicians most commonly (40%; n=27) advising no upper time limit per session. With regards to strength-conditioning exercise during the first trimester, three-fourths (74%; n=50) recommend strength training two to three or four to five days per week, whereas about one-fourth (24%; n=16) do not recommend this type of exercise.

**Table 4:** Physicians' advice regarding the frequency and/or duration with which aerobic and strength-conditioning exercise should be performed during the first trimester

Aerobic exercise frequency (days per week)	%	n
0	1.5	1
1	1.5	1
2-3	45.6	31
4-5	51.5	35
Aerobic exercise duration (minutes per session)		
0	1.5	1
<15	0	0
16-30	33.8	23
>30	25.0	17
no limit	39.7	27
Strength-training frequency (days per week)		
0	23.5	16
1	2.9	2
2-3	55.9	38
4-5	17.6	12

Physicians were asked about the advice given to pregnant patients with no obstetric complications who exercised during the first and second trimesters regarding aerobic and strength-training exercise (duration and intensity) during the third trimester (Table 5). The prevailing recommendation is no change in aerobic or strength-conditioning exercise (57%; n=39 and 41%; n=28, respectively), but many respondents recommend a decrease in aerobic or strength-training activity (25%; n=23 and 32%; n=22), or do not discuss this with their patients (18%; n=12 and 25%; n=17).

**Table 5:** Physicians’ advice to patients who exercised during the first and second trimesters regarding the duration and/or intensity of aerobic and strength-conditioning exercise during the third trimester

Aerobic exercise	%	n
stop all aerobic activity	0	0
decrease aerobic activity	25.0	17
no change in aerobic activity	57.4	39
not addressed with patients	17.6	12
Strength-conditioning exercise		
stop all strength-conditioning exercise	1.5	1
decrease strength-conditioning exercise	32.4	22
no change in strength-conditioning exercise	41.2	28
not addressed with patients	25.0	17

Advice regarding maximum heart rate and perceived exertion

Participants were surveyed on their recommendations regarding maximum heart rate (bpm) and level of perceived exertion during an exercise session (Table 6). More than half (54%; n=37) recommend pregnant patients limit exercise based upon heart rate, most commonly 121-140 bpm (25%; n=17) or 141-160 bpm (24%; n=16), whereas 46% ( n=31) do not advise an upper heart rate limit. Also, more than half of physicians (59%; n=40) believe perceived exertion should be somewhat hard, while one-fourth (27%; n=18) advise no limit to exercise based upon perceived exertion.

**Table 6:** Maximum heart rate and maximum perceived level of exertion physicians recommend pregnant patients achieve during exercise

Maximum heart rate (beats per minute)	%	n
<120	2.9	2
121-140	25.0	17
141-160	23.5	16
161-180	2.9	2
181-200	0	0
no upper limit	45.6	31
Perceived level of exertion		
very, very light	0	0
very light	0	0
fairly light	11.8	8
somewhat hard	58.8	40
hard	2.9	2
very hard	0	0
very, very hard	0	0
no upper limit	26.5	18

Provider knowledge and comfort level

Participants were presented with a list of statements and asked to identify which statement most closely correlates with the 2015 ACOG Committee Opinion regarding exercise and pregnancy. Eighty-eight percent (n=60) of physicians were able to identify the correct ACOG recommendation of unrestricted exercise for women with uncomplicated pregnancies. However, 10% (n=7) believe aerobic and strength-training exercise is recommended only if the maternal heart rate remains less than 140 bpm, and one thinks aerobic and strength-conditioning exercise is contraindicated in all pregnant women. Physicians were asked how comfortable they feel providing advice on exercise during pregnancy: 68% (n=46) report feeling either “comfortable” or “very comfortable” providing recommendations.

Health benefits and need for further research

Providers were presented with a list of health benefits and asked to select the benefits they believe are associated with exercise during an uncomplicated pregnancy (Table 7). Most physicians associate exercise during pregnancy with prevention of excessive weight gain (91%; n=62) and decreased risk of post-partum depression (77%; n=52), gestational diabetes (68%; n=46), and delivering a large for gestational age infant (56%; n=38). Fewer (43%; n=29) believe exercise decreases the duration of labor. All respondents believe there is a need for more research and information regarding the effects of exercise during pregnancy: most (59%; n=40) believe this need is “moderate,” while 29% (n=20) believe it is “slight,” and 12% (n=8) believe it is “severe.”

**Table 7:** Health benefits physicians believe are associated with exercise during an uncomplicated pregnancy 1. GDM= gestational diabetes mellitus 2. LGA= large for gestational age

Benefit	%	n
prevention of excessive weight gain	91.2	62
decreased risk of post-partum depression	76.5	52
decreased risk of GDM^1^	67.6	46
decreased risk of a LGA^2^ infant	55.9	38
decreased duration of labor	42.6	29
risks of exercise outweigh the benefits	2.9	2

## Discussion

Benefits of regular exercise include reduced risk of obesity and diabetes, improved cardiorespiratory fitness, better mood, and a longer life span. Exercise before, during, and after pregnancy reduces the risk of complications during pregnancy and the post-partum period, including low back pain, depressive symptoms, pre-eclampsia, gestational diabetes, preterm delivery, and emergency caesarean section [10-13]. Exercise also provides women a means to prevent excessive weight gain and increases in BMI during pregnancy, which is important because even modest increases in maternal BMI are associated with increased risk of fetal death, stillbirth, and neonatal, perinatal, and infant death [14]. Women who are overweight pre-pregnancy are more likely to have gestational weight gain above the Institute of Medicine’s recommendations [15], so it may be particularly important for this patient population to adopt a regular exercise program.

Despite this evidence, literature suggests many physicians are not providing adequate exercise recommendations to their pregnant patients. One-third of women reported they were not counseled on exercise at their prenatal care appointment in a 2012 study of the general pregnant population in San Francisco [4]. A survey of 188 women between 20-30 weeks gestation by researchers at the University of South Carolina showed similar results [16]. Many pregnant women reported that, unless requested, they received little to no guidance on exercise from their obstetricians in a 2013 study at UNC Chapel Hill. Furthermore, the recommendations provided were often vague and nonspecific beyond being advised "to walk" [5]. A study of overweight and obese pregnant women revealed similar perceptions; notably, these patients were advised to not exercise more intensely than before pregnancy, and since many of these women were sedentary pre-pregnancy, they took this to mean they should continue to not exercise [6]. These findings are problematic because many pregnant women are not meeting the exercise requirements set forth by ACOG, or not exercising at all [7]. However, women are more likely to exercise during pregnancy per the instruction of their physician [9].

With the publication of ACOG’s 2015 Committee Opinion on exercise during pregnancy, this study aims to provide an updated evaluation of providers’ beliefs and recommendations on this subject. The results can be compared to those of Entin and Munhall’s 2006 survey (Table 8), which was interpreted according to the 2002 ACOG Guidelines [2, 3]. Whereas Entin and Munhall found half (52%; n=43) of providers regularly (80-100% of the time) counsel their pregnant patients on exercise, only one-fourth (24%; n=16) of respondents in our study discuss this subject with most (76-100%) of their patients.

 

**Table 8:** Entin and Munhall’s 2006 survey results compared to our survey results

Survey response	Entin & Munhall (2006)	Our results
Do not discuss exercise during pregnancy with most (>75-80% of) patients	52% (n=43)	24% (n=16)
Do not recommend sedentary, healthy pregnant women begin exercising	68% (n=56)	57% (n=39)
Rarely or never recommend strength-training exercise	23% (n=19)	24% (n=16)
Recommend exercise limitations based upon maximum heart rate	62% (n=51)	54% (n=37)
Recommend decreased exercise in the 3rd trimester	54% (n=45)	25% (n=17) and 32% (n=22) for aerobic and resistance exercise, respectively

In both studies, when exercise is discussed, most providers provide suitable advice on aerobic activity. However, their recommendations regarding sedentary pregnant patients, resistance training, maximum heart rate during physical activity, and third trimester exercise are discordant with ACOG’s guidelines. We and Entin and Munhall found that 57% (n=39) and 68% (n=56) of respondents, respectively, do not routinely advise sedentary women with no medical contraindications to initiate a new exercise program during pregnancy. Similarly, we and Entin and Munhall found that 24% (n=16) and 23% (n=19) of respondents, respectively, rarely or never recommend resistance training. It is surprising there has been no significant increase in these recommendations since they are both specifically advocated in the 2015 ACOG Opinion. Regarding maximum heart rate, 54% (n=37) of our and 62% (n=51) of Entin and Munhall’s respondents recommend pregnant patients limit exercise intensity according to this measure, a previous ACOG guideline which was revoked in 1994. Finally, although not an ACOG recommendation, 25% (n=17) and 32% (n=22) of our respondents advise a decrease in aerobic and resistance activity, respectively, in the third trimester. This is an improvement from Entin and Munhall findings that half (54%; n=45) of providers recommend women without obstetric complications reduce these exercises during the last three months of pregnancy.

Regarding the medical benefits of exercise, most respondents to our survey believe exercise helps decrease the risk of GDM, which parallels Entin and Munhall’s findings. Our respondents also believe exercise helps prevent excessive weight gain, post-partum depression, and a large-for-gestational-age infant, but they were more skeptical about exercise decreasing labor duration. Concerning the need for further research about the effects of exercise during pregnancy, most of our respondents believe there is only a “slight” or “moderate” need for more research, whereas most respondents to Entin and Munhall’s survey believed further research is “much needed.” This suggests obstetricians have become more confident in the current understanding of exercise and pregnancy.

Recent literature suggests reasons why physicians are not consistently discussing exercise with their pregnant patients. A 2010 study found obstetricians question the effectiveness of their counseling on nutrition, physical activity and weight gain; they believe pregnant women’s lifestyle habits are more highly influenced by family and cultural factors [17]. Contrary to this belief, physicians’ advice to pregnant patients on these topics has been shown to significantly reduce gestational weight gain and associated health complications [18]. Another possible reason for lack of counseling is that physicians perceive weight and weight gain as sensitive and emotional topics and fear patients may be offended, angered, saddened, or embarrassed by discussing them [17]. Moreover, several studies suggest physicians feel inadequately trained in prenatal counseling on weight gain, nutrition and exercise, and unconfident discussing these topics with their patients [4]. However, this may not explain our results because 68% (n=46) of physicians surveyed report feeling either comfortable or very comfortable advising pregnant women about exercise.

It is unlikely that lack of counseling is due to misconceptions about the benefits of exercise during pregnancy because most obstetricians are able to identify exercise-related health improvements for pregnant women, and nearly all (98%; n=67) believe the benefits of exercise during an uncomplicated pregnancy outweigh the risks (Table 7). Moreover, it is unlikely that inadequate discussion is due to misunderstanding the 2015 ACOG guidelines because 88% (n=60) of physicians accurately report, “Exercise without restriction is recommended” by ACOG.

Lack of time and compensation have been shown to prevent physicians from discussing exercise with pregnant patients [17, 19]. Perhaps the short duration of office visits and the breadth of information physicians feel needs to be covered with patients contributes to their perceived lack of time. A brief video message about diet, exercise and weight gain delivered by an actor-portrayed Video Doctor has been shown to improve exercise and dietary behaviors in pregnant women [20]. This may be one way to increase awareness and shorten the amount of time that physicians spend counseling patients about exercise.  Providing handouts on exercise classes targeted at pregnant women and posting flyers in the waiting rooms is another potential time-saving intervention.

Of great importance, respondents do not routinely advise sedentary women with no medical contraindications to initiate a new exercise program during pregnancy. This mirrors the results of a 2013 study conducted by Penn State researchers on how health care providers counsel overweight and obese pregnant women on physical activity. They found that most overweight and obese pregnant women received limited or no advice on appropriate physical activity during pregnancy. Those who did receive advice were told to not exercise more intensely than before pregnancy; since most of these women were not exercising before they became pregnant, they remained sedentary throughout gestation [6]. These findings are particularly worrisome given a sedentary lifestyle promotes further weight gain and obesity, which increases the risk of severe complications for mother and baby during pregnancy. Thus, one could reasonably argue that it is most critical to prescribe exercise to this patient population above all others.

Reasons for the lack of counseling of sedentary, overweight and obese patients may be similar to those already mentioned regarding the general pregnant population. If physicians question the effectiveness of their advice to the general pregnant population, perhaps they believe patients who lead sedentary lifestyles before pregnancy would be even less receptive to counseling on exercise. Similarly, they may be more reluctant to discuss weight gain and exercise with overweight and obese individuals for fear of embarrassing them. Contrary to this thought, a recent study suggests this patient population is not offended by the discussion of weight and exercise; rather, they want more information on these topics [19]. Unfortunately, we did not ask physicians their beliefs regarding exercise during pregnancy for sedentary, overweight and obese individuals, or their level of comfort discussing exercise with these patients.

## Conclusions

Even though obstetricians believe exercise benefits pregnant women, know it is endorsed by ACOG’s 2015 guidelines, and feel comfortable discussing it with their patients, they are not consistently counseling pregnant patients on this subject. When exercise is discussed, most obstetricians provide suitable advice on aerobic activity; however, their recommendations for previously sedentary patients, resistance training, maximum heart rate during exercise, and third-trimester exercise are discordant with ACOG’s guidelines. Future research should focus further on clarifying the barriers preventing physicians from discussing exercise during pregnancy, and implementing effective solutions to this problem. Further, studies assessing physicians’ beliefs about sedentary, overweight and obese individuals increasing their physical activity during gestation, and their level of comfort recommending this, would help elucidate why they are failing to discuss exercise with these patients.

## Appendices

Survey on Advice Given to Pregnant Patients Regarding Exercise during Pregnancy

1. Do you care for women during pregnancy?

a. Yes

b. No

2. What description best characterizes your provider level?

a. Physician Assistant

b. Nurse Practitioner

c. Nurse

d. Physician

3. For how many years have you practiced in the field of obstetrics?

a. Less than 5 years

b. 6-10 years

c. 11-15 years

d. 16-20 years

e. More than 20 years

4. What is your gender?

a. Male

b. Female

5. In your opinion, which of the following statements most closely correlates with the 2015 American College of Obstetrics and Gynecology (ACOG) Committee Opinion regarding exercise and pregnancy?

a. Aerobic and strength-conditioning exercises are not recommended during an uncomplicated pregnancy

b. Aerobic and strength-training exercises are recommended as long as the maternal heart rate remains less than 140 beats per minute

c. Women with uncomplicated pregnancies should be encouraged to engage in aerobic and strength-conditioning exercises throughout pregnancy and postpartum.

d. Aerobic and strength-conditioning exercises without restriction are recommended for all pregnant women.

6. With what percentage of your pregnant patients do you specifically address the issue of exercise during pregnancy?

a. 0-25%

b. 26-50%

c. 51-75%

d. 76-100%

7. Ordinarily, how do you initiate discussion about exercise during pregnancy with your pregnant patients?

a. I do not discuss exercise during pregnancy with my patients

b. I initiate the discussion about exercise during pregnancy with my patients

c. I discuss exercise during pregnancy only if the patient asks for my input

d. My staff discusses exercise during pregnancy with my patients

8. When discussing exercise during the first trimester, how often do you suggest that your patients participate in aerobic exercise such as stationary biking, jogging, swimming etc.?

a. Never. I do not recommend exercise during pregnancy.

b. 1 day per week

c. 2-3 days per week

d. 4-5 days per week

9. When discussing exercise during the first trimester, what duration do you typically recommend for aerobic exercise?

a. I do not recommend aerobic exercise during the first trimester of pregnancy.

b. Less than 15 minutes of continuous exercise

c. 16-30 minutes of continuous exercise

d. More than 30 minutes of continuous exercise

e. I do not set a limit on the duration of continuous exercise

10. When discussing exercise during the first trimester, how often do you suggest that your patients participate in strength-conditioning exercises?

a. I do not recommend strength-conditioning exercise during the first trimester of pregnancy.

b. 1 day per week

c. 2-3 days per week

d. 4-5 days per week

11. For your pregnant patients with no obstetric complications who have exercised during the first and second trimesters, what recommendation do you give regarding aerobic exercise (duration and/or intensity) during the third trimester?

a. I recommend that patients suspend all aerobic activity during the third trimester

b. I recommend that patients decrease aerobic activity during the third trimester

c. I recommend no change in the amount of aerobic activity during the third trimester

d. I do not address changing aerobic exercise (duration and/or intensity) during the third trimester

12. For your pregnant patients with no obstetric complications who have exercised during the first and second trimesters, what recommendation do you give regarding strength-conditioning (duration and/or intensity) during the third trimester?

a. I recommend that patients suspend all strength-conditioning during the third trimester

b. I recommend that patients decrease strength-conditioning during the third trimester

c. I recommend no change in strength-conditioning during the third trimester

d. I do not address strength-conditioning (duration and/or intensity) during the third trimester

13. When discussing exercise with your pregnant patients, what is the maximum heart rate that you recommend your patients achieve during exercise?

a. <120 bpm

b. 121-140 bpm

c. 141-160 bpm

d. 161-180 bpm

e. 180-200 bpm

f. I do not advise my patients to limit exercise intensity based on heart rate

14. When discussing exercise with your pregnant patients, what level of perceived exertion do you recommend patients achieve during exercise?

a. Very, very light

b. Very light

c. Fairly light

d. Somewhat hard

e. Hard

f. Very hard

g. Very, very hard

h. I do not advise my patients to limit exercise intensity based on perceived exertion

15. For your sedentary pregnant patients who have no medical reason to avoid exercise, how often do you recommend beginning a new exercise program during pregnancy?

a. I never recommend that these patients begin an exercise program during pregnancy

b. I rarely recommend that these patients begin an exercise program during pregnancy

c. I sometimes recommend that these patients begin an exercise program during pregnancy

d. I usually recommend that these patients begin an exercise program during pregnancy

e. I always recommend that these patients begin an exercise program during pregnancy

16. What role do you believe a regular exercise program has on the probability of a patient developing gestational diabetes mellitus (GDM)?

a. A regular exercise program somewhat reduces the probability of developing GDM

b. A regular exercise program greatly reduces the probability of developing GDM

c. A regular exercise program has no effect on the probability of developing GDM

d. A regular exercise program increases the probability of developing GDM

e. I am uncertain of the correlation between exercise and GDM

17. What risks are associated with exercise in uncomplicated pregnancy? (circle all that apply)

a. There are no risks associated with exercise in uncomplicated pregnancy

b. Miscarriage

c. Preterm labor

d. Low birth weight

e. Cervical insufficiency

f. Hyperthermia

g. Pregnancy induced hypertension

h. Other ___________________________________________________

18. What are benefits do you believe are associated with exercise in uncomplicated pregnancy? (circle all that apply)

a. The risks of exercise outweigh the benefits in uncomplicated pregnancy

b. Prevent excessive weight gain

c. Decreased duration of labor

d. Decreased risk of post-partum depression

e. Decreased risk of delivering a large for gestational age infant

f. Decrease risk of gestational diabetes

g. Other _____________________________________________________________

19. How much of a need do you believe exists for more research and information regarding the effects of exercise during pregnancy on pregnancy outcomes?

a. No need

b. Slight need

c. Moderate need

d. Severe need

20. How comfortable do you feel providing advice on exercise during pregnancy?

a. Not comfortable

b. Slightly comfortable

c. Comfortable

d. Very comfortable
